# SD-GASNet: Efficient Dual-Domain Multi-Scale Fusion Network with Self-Distillation for Surface Defect Detection

**DOI:** 10.3390/s26010023

**Published:** 2025-12-19

**Authors:** Jiahao Fu, Zili Zhang, Tao Peng, Xinrong Hu, Jun Zhang

**Affiliations:** 1 School of Computer Science and Artificial Intelligence, Wuhan Textile University, Wuhan 430200, China; 2315363128@wtu.edu.cn (J.F.); pt@wtu.edu.cn (T.P.); hxr@wtu.edu.cn (X.H.); 2 Hubei Province Clothing Informatization Technology Research Center, Wuhan 430200, China; 3 School of Computer Science and Engineering, Wuhan Institute of Technology, Wuhan 430205, China; zjun@wit.edu.cn

**Keywords:** surface defect detection, sensor-based industrial applications, frequency domain information, knowledge distillation, fusion network

## Abstract

Surface defect detection is vital in industrial quality control. While deep learning has largely automated inspection, accurately locating defects with large-scale variations or those difficult to distinguish from similar backgrounds remains challenging. Furthermore, achieving high-precision and real-time performance under limited computational resources in deployment environments complicates effective solutions. In this work, we propose SD-GASNet, a network based on a self-distillation model compression strategy. To identify subtle defects, we design an Alignment, Enhancement, and Synchronization Feature Pyramid Network (AES-FPN) fusion network incorporating the Frequency Domain Information Gathering-and-Allocation (FIGA) mechanism and the Channel Synchronization (CS) module for industrial images from different sensors. Specifically, FIGA refines features via the Multi-scale Feature Alignment (MFA) module, then the Frequency-Guided Perception Enhancement Module (FGPEM) extracts high- and low-frequency information to enhance spatial representation. The CS module compensates for information loss during feature fusion. Addressing computational constraints, we adopt self-distillation with an Enhanced KL divergence loss function to boost lightweight model performance. Extensive experiments on three public datasets (NEU-DET, PCB, and TILDA) demonstrate that SD-GASNet achieves state-of-the-art performance with excellent generalization, delivering superior accuracy and a competitive inference speed of 180 FPS, offering a robust and generalizable solution for sensor-based industrial imaging applications.

## 1. Introduction

**Figure 1:**
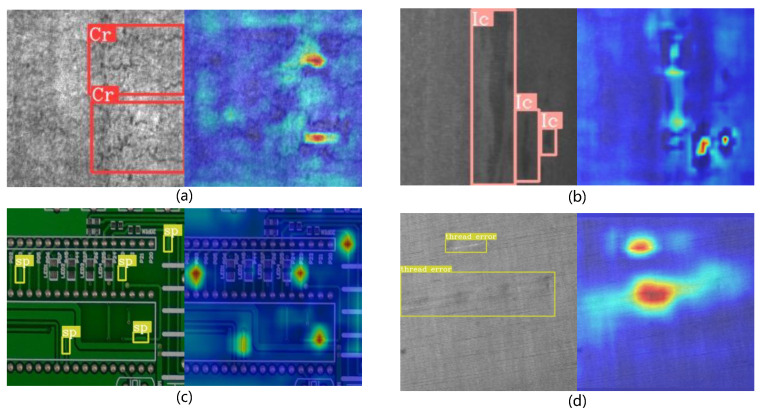
Examples of industrial surface defects that exhibit high similarity to the background and significant scale variations. In each image pair, the left panel shows the ground-truth defect mask, while the right panel displays the corresponding heatmap, where red regions indicate higher attention values). (**a**) and (**b**) show two types of steel defects: crazing and inclusion, respectively. (**c**) depicts a spur defect on a PCB. (**d**) illustrates a fabric defect resulting from a material flaw.

Surface defects on industrial products arise from a variety of sources, including production-related anomalies in hot-rolled steel, material flaws or contamination in fabrics, and corrosion on printed circuit boards (PCBs). These characteristics distinguish surface defect detection in industrial contexts from traditional object detection tasks, making it a uniquely challenging problem. Specifically, background textures may be similar to potential defects, making it very difficult to accurately identify these defects. Meanwhile, process fluctuations during manufacturing further exacerbate the diversity and uncertainty in defect scale and shape. In addition, industrial production lines typically require real-time detection capabilities, which require models to achieve high inference speed while maintaining low computational and memory overhead. In summary, the detection of surface defects in industrial scenarios is primarily faced with three key issues:

- The inapparent nature of the defects makes them difficult to distinguish from complex backgrounds, as illustrated in Figure 1a,c,d.
- The size of the defect varies considerably, as shown in Figure 1b.
- In real-world industrial production environments, achieving a balance between high detection precision and high inference speed becomes particularly challenging due to the limitations of available computational resources.

Previous methods used mainly traditional feature extraction techniques [1], such as threshold segmentation [2] and pattern recognition [3], to achieve defect classification and localization. These methods have made significant contributions to industrial defect detection. Recently, with the development of deep learning and image processing techniques, more and more researchers are adopting deep learning techniques for defect detection [4,5,6].

Fusion networks designed for general object detection were not suitable for defect detection. Many studies [7,8] have designed specialized fusion networks for defect samples to improve feature representation. These fusion models aim to extract deeper defect features to better distinguish low-contrast defect samples from the background. However, a significant limitation persists in their handling of large-scale variations, primarily due to two interconnected issues. First, they often lack direct information exchange between non-adjacent feature layers, preventing the effective propagation of multi-scale context [9]. Second, insufficient fusion of multi-scale features across disparate receptive fields constrains the network’s capacity to concurrently capture both fine-grained and large-scale defects.

Some existing works have recognized this dilemma and designed effective fusion networks. For example, Gao et al. [10] proposed the Feature Collection and Compression Network (FCCN) fusion module to expand the receptive field and collect multi-scale feature information. In Libra R-CNN, Pang et al. [11] proposed the Balanced Feature Pyramid (BFP) to achieve a more balanced fusion of multi-level features. Both FCCN and BFP methods recognized the importance of information interaction between non-adjacent feature layers. However, these methods simply distribute cross-layer information proportionally to each layer, inevitably introduces irrelevant noise and results in information loss, which can significantly affect their efficiency.

Furthermore, defects can be viewed as abrupt gray-level perturbations against the background; due to low contrast, such perturbations are barely perceptible in the spatial domain. In the frequency [12] domain, the image is decomposed into sinusoidal components of different frequencies, enabling these high-frequency perturbations to be easily detected. For example, FcaNet [13] replaced GAP with multi-frequency DCT coefficients to enrich channel attention, while Frank et al. [14] exploited DCT-exposed upsampling artifacts for linear deep-fake detection.

Therefore, to address the aforementioned challenges and limitations, we propose the Frequency Domain Information Gathering-and-Allocation (FIGA) mechanism and the Channel Synchronization (CS) module within our proposed Alignment, Enhancement, and Synchronization Feature Pyramid Network (AES-FPN). FIGA aggregates multi-level information through the Multi-scale Feature Alignment (MFA) module and the Frequency-Guided Perception Enhancement Module (FGPEM). Among these, MFA mainly targets the issue of insufficient cross-layer information exchange. To enhance the perception of defects, FGPEM introduces our proposed Position-based DCT-II (PDCT) filter. This filter, which builds upon the standard Discrete Cosine Transform (DCT) [15] with a novel position-guided selection strategy, separates frequency domain information into two key components: high-frequency signals corresponding to boundary details and low-frequency signals representing texture information. The CS module is designed mainly to handle potential information loss during the fusion process.

Current defect detection systems face a major challenge, as they often rely on massive computing resources and storage space to achieve optimal performance [16,17]. To balance the accuracy and speed of defect detection models, especially in resource-constrained application scenarios, model compression techniques like knowledge distillation are widely adopted [18,19]. However, traditional knowledge distillation methods face two main limitations, first, the selection of an optimal teacher model is non-trivial, second, student models with fewer parameters trained through this approach typically struggle to surpass the performance of the teacher model.

Inspired by the above work, this paper proposes an efficient defect detection method called Efficient Dual-Domain Multi-Scale Fusion Network with Self-Distillation (SD-GASNet) which can satisfy the requirement of high speed and high accuracy for industrial defect detection. Specifically, our contributions are as follows:

- We propose a novel FGPEM built upon a PDCT filter to effectively capture frequency-domain features, thereby reinforcing defect representation in the spatial domain.
- We introduce AES-FPN, an efficient multi-scale feature fusion network that leverages a FIGA mechanism to handle scale and background challenges, and a CS module to preserve channel-wise completeness of effective information.
- We design an efficient self-distillation framework, consisting of an enhanced KL-divergence loss and a novel SD-Head, which boosts performance without the need for an external teacher model.
- We develop SD-GASNet, a highly efficient network that achieves a state-of-the-art balance between speed and accuracy across three public datasets.

The rest of this article is organized as follows. In Section 2 we briefly introduce the related work. Section 3 describes our network. The experimental settings and results are presented and analyzed in Section 4. Finally, we conclude the findings in Section 5.

## 2. Related Work

In recent years, with the rapid development of deep learning, deep learning-based defect detection methods have made significant progress. This section presents a review of relevant studies across three domains: deep learning-based defect detection (Section 2.1), feature fusion strategies in defect detection (Section 2.2), and knowledge distillation techniques (Section 2.3).

### 2.1. Deep-Learning-Based Defect Detection

Wang et al. [20] proposed the real-time detection network, which was based on the ResNet-DCN lightweight modular encoder-decoder network, and designed the skip connection and pyramid feature fusion modules to enhance detection accuracy. Luo et al. [21] added feature enhancement and auxiliary localization modules to the classic Faster-RCNN [22] two-stage object detection model, which obtained the superior result on the FPCB dataset. ETDNet [4] employed MLVT as its backbone and introduced a Channel-Modulated Feature Fusion Network (CM-FPN) to effectively integrate global and local features, which demonstrated outstanding performance across various defect detection dataset. Recent studies have also explored generative models for this task; for instance, Hu et al. [23] utilized an adversarial autoencoder to learn representations from pencil-lead-break-induced ultrasonic signals for robust rail damage detection. Due to the fast and efficient characteristics of the YOLO [24] series detectors, many studies [25,26,27] have explored their application in the field of defect detection.

### 2.2. Feature Fusion in Defect Detection

To address the significant issue of scale variations in defect images, some research optimizes the feature fusion module of the detection network. To address the challenge of preserving weak defect features, STMA-Net [28] proposed a multi-level attention feature fusion network that uses a multi-scale channel attention module to guide the process. Zhao et al. [29] proposed a Multi-scale Feature Fusion module (MFF) based on the attention mechanism [30]. MFF effectively enhanced the representation of defect features by introducing two types of attention module and made it easier to distinguish defects from the background. AFF-Net [31] integrated a Swin Transformer backbone with a focused feature pyramid fusion network, achieving competitive performance on steel defect datasets. To address the challenge of accurately identifying small defects on PCB dataset, YOLO-HMC [32] incorporated an improved multiple convolutional block attention module and used the content-aware reassembly of features for feature reorganization to achieve positive results.

These studies have optimized feature fusion modules for various defect detection tasks, such as steel and fabric defect detection. They mainly strengthen feature representation to improve fusion efficiency. In contrast, our research takes a different approach. Since the VIT-based backbone already extracts highly representative features, our fusion module effectively uses these features and prevents information loss during the fusion process. Some studies share similar ideas with ours. LLD-MFCOS [33] proposed the Cross-layer Refined Feature Pyramid Network (CR-FPN), which incorporated cross-layer information into the lateral connections of the FPN [34] and enhanced feature fusion by utilizing the hybrid non-local module. SDDNet [35] extracted multi-scale features by exploiting Feature Retention Blocks (FRB) and Skip-connected Dense Connection Modules (SDCM). The FRB addressed the loss of detailed information caused by down-sampling, while the SDCM facilitated the propagation of fine-grained features to higher-level layers.

### 2.3. Knowledge Distillation

Knowledge distillation makes use of the high-performance model (teacher) to guide the training of student model. By transferring knowledge to the student model, it enables more efficient learning. This method has demonstrated broad application prospects in various fields. In order to address the problem of insufficient labels in classification tasks, Hinton et al. [36] first applied knowledge distillation techniques to classification tasks. Subsequently, Chen et al. [37] first apply knowledge distillation to multi-class object detection.

In real-world industrial environments, there are high demands for model inference speed. Knowledge distillation, as a feasible model compression method, has been widely applied in the field of defect detection. CADN [38] took advantage of heatmaps as a knowledge representation, designed novel transfer modules, and employed Mean Squared Error (MSE) as a distillation loss function to conduct knowledge distillation. Cao et al. [39] proposed informative knowledge distillation to mitigate overfitting in image anomaly segmentation. Their approach integrated multi-hierarchical knowledge distillation, multi-hierarchical anomaly score fusion, and contextual similarity loss.

Although knowledge distillation has brought performance improvements in these works, it is not universally applicable due to two difficulties: first, it is challenging to accurately define the teacher model as a guide, second, the performance of student model often fails to reach the level of teacher model. To address these challenges, we propose a self-distillation training strategy that eliminates the need for an additional teacher model and improves performance by its internal distillation mechanism.

## 3. The Proposed SD-GASNet Method

**Figure 2:**
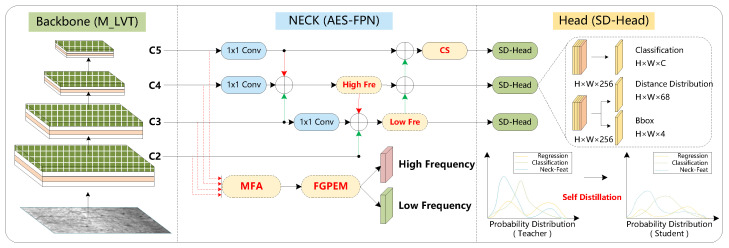
Overall architecture of the proposed SD-GASNet. The model consists of three main components: (1) the MLVT backbone for global feature extraction; (2) the AES-FPN neck that performs multi-scale feature fusion by integrating our FIGA mechanism–including the MFA, FGPEM, and Inject modules– with the CS module; and (3) the self-distillation head that employs an enhanced KL divergence loss to improve performance.

This section introduces the proposed SD-GASNet, whose overall architecture is shown in Figure 2. The following subsections describe its key components in detail: the FIGA mechanism (Section 3.1), the AES-FPN fusion network (Section 3.2), the detector architecture (Section 3.3), and the loss function (Section 3.4).

### 3.1. Frequency Domain Information Gathering-And-Allocation Mechanism

**Figure 3:**
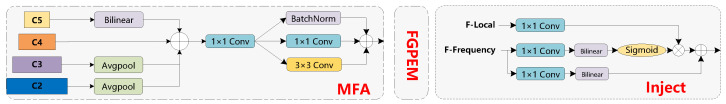
Frequency Domain Information Gathering-and-Allocation mechanism consists of three parts, includes Multi-scale Feature Alignment module, Frequency-Guided Perception Enhancement module, and an information Inject module.

**Figure 4:**
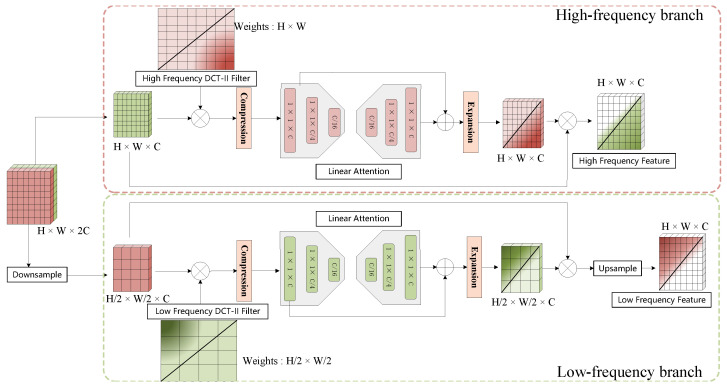
Architecture of the FGPEM module. The module processes aggregated multi-level features through two parallel branches: a high-frequency branch and a low-frequency branch. Firstly, in each branch, features are processed by a corresponding PDCT filter and an MLP-based linear attention block. Secondly, the outputs of both branches are independently fused with the original features via a residual connection to enhance representation.

The backbone generates four feature maps at different resolutions (1/4, 1/8, 1/16, and 1/32 of the original image resolution). These feature maps are associated with defects of different scales, high-level maps (e.g., 1/16 and 1/32) are better suited for detecting larger defects and offering a more macroscopic distinction between defects and backgrounds. Low-level maps (e.g., 1/4 and 1/8) provide finer details to capture smaller defects and intricate texture information. The MFA module aims to address the issue of insufficient cross-layer information interaction in traditional fusion networks. The FGPEM module utilizes a PDCT filter to extract high-frequency features (corresponding to boundary information) and low-frequency features (corresponding to texture information), thereby enhancing spatial feature representation and consequently improving detection performance. Figure 3 shows the overall structure of the FIGA mechanism, while Figure 4 provides a detailed view of the FGPEM module.

To achieve an initial fusion of multi-scale features extracted from different levels *i* of the backbone network, we first align these features to a common spatial resolution. Let $C_{i}\in R^{H_{i}\times W_{i}\times D_{i}}$ denote the feature map from the *i*-th level, where $H_{i},W_{i},$ and $D_{i}$ are its height, width, and channel depth, respectively. We designate the feature map $C_{4}$ as the reference, and other feature maps ($C_{2},C_{3},$ and $C_{5}$) are spatially resampled to match the spatial dimensions $\left(H_{4},W_{4}\right)$ of $C_{4}$. The spatially aligned feature map for level *i*, denoted as $C_{i}^{A}\in R^{H_{4}\times W_{4}\times D_{i}}$, is obtained by applying a level-specific resampling operator $R_{i}$:

(1) $$C_{i}^{A}=R_{i}\left(C_{i}\right)$$

(2) $$R_{i}\left(X\right)=\left\{\begin{array}{cc} {\mathrm{Upsample}}_{\mathrm{BL}}\left(X,\left(H_{4},W_{4}\right)\right) & \mathrm{if}\;i>4\\ X & \mathrm{if}\;i=4\\ {\mathrm{Downsample}}_{\mathrm{AP}}\left(X,\left(H_{4},W_{4}\right)\right) & \mathrm{if}\;i<4 \end{array}\right.$$

Here, ${\mathrm{Upsample}}_{\mathrm{BL}}\left(\cdot ,\mathrm{target}\_\mathrm{dims}\right)$ denotes an upsampling operation to target_dims using bilinear interpolation. Conversely, ${\mathrm{Downsample}}_{\mathrm{AP}}\left(\cdot ,\mathrm{target}\_\mathrm{dims}\right)$ represents a downsampling operation to target_dims using adaptive average pooling. The set of features ${\left\{C_{i}^{A}\right\}}_{i\in \{2,3,4,5\}}$ are now aligned to the same spatial resolution. Subsequently, these resolution-aligned features are concatenated along the channel dimension to produce a unified multi-scale feature representation $F_{\mathrm{fused}}$ as follows:

(3) $$F_{\mathrm{fused}}=\mathrm{Concat}\left(C_{2}^{A},C_{3}^{A},C_{4}^{A},C_{5}^{A}\right)$$

To further refine the aligned features, the tensor $F_{\mathrm{fused}}\in R^{H\times W\times D}$ is passed through the core enhancement stage of our Multi-scale Feature Alignment (MFA) module, which consists of a cascade of three RepVGG [40] blocks. Each block enhances features using a training-time multi-branch structure. This structure involves concatenating outputs from a 1×1 convolution ($\phi_{1\times 1}$), a Batch Normalization (BN) layer applied to the output of an initial 1×1 convolution, and a 3×3 convolution ($\phi_{3\times 3}$). Critically, this multi-branch setup fuses into a single 3×3 convolution during inference, thereby improving feature representation without increasing inference cost. The operations are defined as:

(4) $$O_{R}\left(X\right)=\mathrm{Concat}\left(\phi_{1\times 1}\left(X\right),\mathrm{BN}\left(\phi_{1\times 1}\left(X\right)\right),\phi_{3\times 3}\left(X\right)\right)$$

(5) $$X_{k}=O_{R}\left(X_{k-1}\right)\;\mathrm{for}\;k=1,\ldots ,3;\;X_{0}=F_{\mathrm{fused}}$$

As shown in Figure 4, after MFA alignment, the features are further processed through FGPEM for frequency domain feature extraction. The extracted frequency domain features are embedded with the original spatial features, thereby enhancing their perception capability of the defect samples.

DCT-II transforms images from the spatial domain to the frequency domain. In JPEG image compression, high-frequency components obtained through DCT-II are typically discarded to achieve efficient data compression. In the frequency coefficient matrix, the coefficients in the top-left corner represent low-frequency information (such as overall defect texture information), while coefficients in the bottom-right corner represent high-frequency information (such as defect edge information). This paper filters the DCT-II basis functions based on preset position, named PDCT, to selectively extract either high-frequency or low-frequency coefficients. The specific formula for constructing the PDCT filter element $W_{c,t_{x},t_{y}}$ is as follows:

(6) $$W_{c,t_{x},t_{y}}=\left\{\begin{array}{cc} \frac{\cos\left(\frac{\pi \cdot u_{x}\cdot \left(t_{x}+0.5\right)}{D_{w}}\right)\cos\left(\frac{\pi \cdot v_{y}\cdot \left(t_{y}+0.5\right)}{D_{h}}\right)}{\sqrt{D_{w}}\sqrt{D_{h}}} & \mathrm{if}\;u_{x}=0\;\mathrm{and}\;v_{y}=0\\ \frac{\cos\left(\frac{\pi \cdot u_{x}\cdot \left(t_{x}+0.5\right)}{D_{w}}\right)\cos\left(\frac{\pi \cdot v_{y}\cdot \left(t_{y}+0.5\right)}{D_{h}}\right)}{\sqrt{D_{w}}\sqrt{D_{h}}}\cdot 2 & \mathrm{if}\;u_{x}>0\;\mathrm{or}\;v_{y}>0 \end{array}\right.$$

Here, $W_{c,t_{x},t_{y}}$ represents the intensity of the selected 2D DCT-II basis function at spatial position $\left(t_{x},t_{y}\right)$ for a specific channel *c*. This 2D basis function is constructed by the product of two one-dimensional DCT cosine basis functions: one corresponding to the horizontal direction (parameterized by $u_{x}$ and $t_{x}$) and the other to the vertical direction (parameterized by $v_{y}$ and $t_{y}$). $D_{w}$ and $D_{h}$ are the width and height of the filter, respectively. The term $\left(t_{x}+0.5\right)$ accounts for sampling at pixel centers, which is a characteristic feature of the DCT-II transform. The normalization factor $\frac{1}{\sqrt{D_{w}D_{h}}}$ ensures the orthogonality of the basis functions. Conditional multiplication by $\sqrt{2}$ distinguishes the DC component ($u_{x}=0$ and $v_{y}=0$) from the AC components ($u_{x}>0$ or $v_{y}>0$), which is a standard normalization for DCT-II.

After defining the filter elements, the frequency response $R_{b,c}$ for each channel is obtained by summing the element-wise product of the input feature map $X_{b,c,t_{x},t_{y}}$ and the constructed filter $W_{c,t_{x},t_{y}}$:

(7) $$R_{b,c}=\sum_{t_{x}=0}^{D_{w}-1}\sum_{t_{y}=0}^{D_{h}-1}X_{b,c,t_{x},t_{y}}\cdot W_{c,t_{x},t_{y}}$$

The frequency index pair $\left(u_{x},v_{y}\right)$ for each channel *c* is determined by a preset position-based frequency selection strategy. Specifically, $\left(u_{x},v_{y}\right)$ is derived from initial fixed points $u_{x}^{\mathrm{initial}}$ and $v_{y}^{\mathrm{initial}}$, which are the horizontal and vertical indices drawn from a specific, pre-defined set of 8 frequency components on a 10×10 grid. Our selection comprises 4 low-frequency components with coordinate pairs (0,0), (0,1), (1,0), and (1,1), and 4 high-frequency components with coordinate pairs (9,9), (9,8), (8,9), and (8,8). For any given frequency component selected, $u_{x}^{\mathrm{initial}}$ takes its horizontal coordinate value while $v_{y}^{\mathrm{initial}}$ takes its vertical coordinate value. These initial indices are then scaled by the filter dimensions:

(8) $$u_{x}=u_{x}^{\mathrm{initial}}\times \left\lfloor D_{h}/10\right\rfloor$$

(9) $$v_{y}=v_{y}^{\mathrm{initial}}\times \left\lfloor D_{w}/10\right\rfloor$$

After obtaining the frequency response $R_{b,c}$ for the corresponding channel, the channel attention weights are calculated to enhance the representation of the original spatial domain features. This process involves a function $F_{\mathrm{Attn}}$ that takes $R_{b,c}$ as input:

(10) $$F_{\mathrm{Attn}}\left(R_{b,c}\right)=\sigma \left(W_{2}\left(\mathrm{ReLU}\left(W_{1}R_{b,c}+b_{1}\right)\right)+b_{2}\right)$$

Here, $F_{\mathrm{Attn}}\left(R_{b,c}\right)$ is the calculated channel attention weight for channel *c* in batch *b*, which is derived from $R_{b,c}$ using two linear layers ($W_{1},b_{1},W_{2},b_{2}$) with ReLU and σ activations. The final output feature map $Y_{b,c,h,w}$ is then obtained by element-wise multiplication of the original input feature map $X_{b,c,h,w}$ at spatial position (h,w) with these attention weights:

(11) $$Y_{b,c,h,w}=X_{b,c,h,w}\cdot F_{Attn}\left(R_{b,c}\right)$$

### 3.2. AES-FPN

Recently, ASFF [41] and PAFPN [42] have improved the performance of multi-scale object detectors by using lateral connections to fuse features from different levels. These studies reveal the complementarity between shallow and deep features in the network. However, they focus on information interaction between adjacent levels, with less consideration for feature interaction between non-adjacent levels. Some studies, such as BFP [11] and CR-FPN [33], have already made improvements to address the issue of insufficient cross-layer information interaction.

It can be recognized that detectors can effectively detect large defects, while small objects often exhibit poorer detection performance due to their limited feature information. Inspired by previous work, we design a new fusion network called AES-FPN to improve the feature fusion process. As shown in Figure 2, the AES-FPN module enhances multi-scale feature fusion through two key innovations: the FIGA mechanism and the CS module. The FIGA mechanism is designed to extract frequency-domain insights. It generates two types of enhanced spatial features: one enriched with high-frequency boundary information (corresponding to defect edges) and the other with low-frequency texture information (capturing defect patterns). Subsequently, these specialized features are selectively injected into the fusion network to maximize their impact. The high-frequency features are fed into the upper layers of the model to strengthen the defect recognition, while the low-frequency features are directed to the lower layers of model to improve the distinction between defects and the background. The CS module is designed to prevent information loss during this fusion process.

The process of embedding the global frequency information into local features is accomplished by Inject operation. This operation takes two sets of inputs: the high-frequency ($H_{f}$) and low-frequency ($L_{f}$) global features provided by the FGPEM module, and the local feature maps ($C_{4}^{\prime }$ and $C_{3}^{\prime }$) targeted for enhancement.

(12) $$Inject(C_{l},F_{g})=\phi_{L}\left(C_{l}\right)⊙G(F_{g},C_{l})+\phi_{G}(F_{g},C_{l})$$

(13) $$C_{4}^{t}=Inject\left(C_{4}^{\prime },H_{f}\right)$$

(14) $$C_{3}^{t}=Inject\left(C_{3}^{\prime },L_{f}\right)$$

(15) $$C_{4}^{\prime }=C_{4}+↑C_{5}+↓C_{3}$$

(16) $$C_{3}^{\prime }=C_{3}+↑C_{4}^{t}+↓C_{2}$$

**Figure 5:**
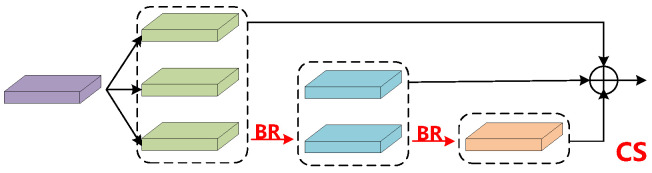
Structure of the Channel Synchronization module. The information between input feature channels is fully exploited through a three-layer stacked BR module.

In Formula (12), $C_{l}$ represents target local features and $F_{g}$ denotes auxiliary global features. $G(F_{g},C_{l})$ signifies a gating mechanism. In the output component, the output of the third feature $C_{3}^{out}$ is obtained by embedding the low frequency auxiliary information $L_{f}$ into $C_{3}$. The output of the fourth layer $C_{4}^{out}$ is obtained by injecting the high frequency auxiliary information $H_{f}$ into $C_{4}$ and then fusing it with $C_{3}^{out}$. The output of the fifth feature layer $C_{5}^{out}$ is computed by first fusing $C_{5}$ with $C_{4}^{out}$, and then applying a CS operation, as shown in Figure 5.

(17) $$C_{3}^{out}=C_{3}^{t}$$

(18) $$C_{4}^{out}=C_{4}^{t}+↓C_{3}^{out}$$

(19) $$C_{5}^{out}=CS\left(C_{5}+↓C_{4}^{out}\right)$$

**Figure 6:**
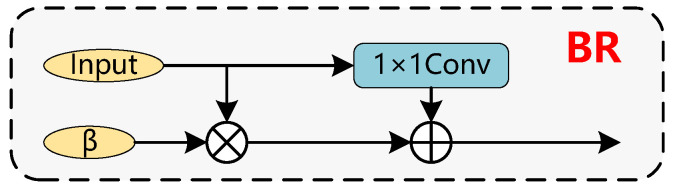
Structure of the Bottle Rep. The learnable parameter β selectively distinguishes important information from irrelevant information during the training process.

The CS module ensures the synchronization between the information obtained from the fusion network and the feature extraction network, preventing the loss of channel information during feature fusion. It can be observed that there is a loss of feature information in the feature fusion component when $C_{5}$ is fused with the downsampled $C_{4}^{out}$, as $C_{5}$ does not inject any auxiliary information. Information loss will affect detection performance, so this paper proposes the CS module shown in Figure 5 to solve this problem. As illustrated, the input first passes through a 1×1 convolution module, increasing the channel dimension to 384, then split it into three equal parts. At this stage, most of the feature information in the channels cannot be utilized directly and required further processing. As shown in Figure 6, Bottle Rep (BR) module is used to extract channel information from each part. Specifically, the BR module introduces a learnable parameter β as a metric to assess information significance, which is used to control the scaling of channel. After three rounds of extraction through the BR module, the resulting features are concatenated along the channel dimension and then adjusted to match the dimension of the backbone network using a final 1×1 convolution operation. After a single CS operation, $C_{5}^{out}$ successfully recovers the lost channel information. The formula for the CS module is expressed as follows:

(20) $$\mathrm{BR}\left(Z\right)=Z+\beta \cdot {\mathrm{Conv}}_{1\times 1}\left(\sigma \left({\mathrm{Conv}}_{3\times 3}\left({\mathrm{Conv}}_{1\times 1}\left(Z\right)\right)\right)\right)$$

σ denotes the ReLU activation function, β∈R, and *C* is a channel-wise learnable scaling factor (normalized to [0,1] via a sigmoid function), which controls the information retention ratio. First, the channels are compressed by a 1×1 convolution (dimensionality reduction to C/4), then spatial features are extracted via a 3×3 convolution, and finally, the channel number is restored by a 1×1 convolution.

(21) $$Y=\mathrm{Concat}\left({\left[\mathrm{BR}\left(X_{i},\beta_{i}\right)\right]}_{i=1}^{3}\right),\;X_{i}=\mathrm{Split}\left({\mathrm{Conv}}_{1\times 1}^{↑}\left(X\right)\right)$$

(22) $$Y_{\mathrm{out}}={\mathrm{Conv}}_{1\times 1}\left(Y\right)$$

The Channel Split (CS) core consists of two steps: split-enhance-concatenate and dimensionality reduction. Equation (21) represents splitting along the channels and enhancing channel expressive power through the BR module. Equation (22) represents the concatenation and restoration operations. Here, ↑ denotes up-dimensioning to 384, and ↓ denotes down-dimensioning to the backbone network’s channel number. The BR module dynamically recovers lost channel information via the β parameter, and the CS module enhances feature expression through concatenation.

### 3.3. SD-GASNet Detector

SD-GASNet adopts the lightweight and efficient MLVT [4] to extract feature and uses the proposed AES-FPN to fuse the feature of cross-layer multi-scale. In the detector head, an enhanced KL divergence loss function is designed for self-distillation training, which further improves the detector performance without an additional teacher model and achieves a balance between high performance and fast speed.

In the multi-scale feature fusion module of SD-GASNet, the AES-FPN module incorporates FIGA mechanism along with CS module. The FIGA mechanism employs MFA and FGPEM to align and fuse multi-scale frequency feature, which is then embedded into the shallow feature layers as auxiliary information. The CS module addresses the issue of channel information loss by controlling the scaling of high-level feature channels through a learnable parameter β.

In addition, SD-GASNet employs a decoupled self-distillation head, which introduces DFL [43] to assist in the regression of target boxes and implements self-distillation based on an enhanced KL divergence loss. The features fused by AES-FPN fusion network are also included in the distillation process to alleviate the phenomenon of knowledge loss encountered by the student network during training. Through this distillation method, the performance of model is self-improved without an external model.

GASNet is initially trained using the same method without the distillation branch. The pre-trained model subsequently serves as the “teacher” model in the self-distillation framework. During this distillation, features from the outputs of both the AES-FPN fusion network and the self-distillation head are utilized to enhance training.

**Figure 7:**
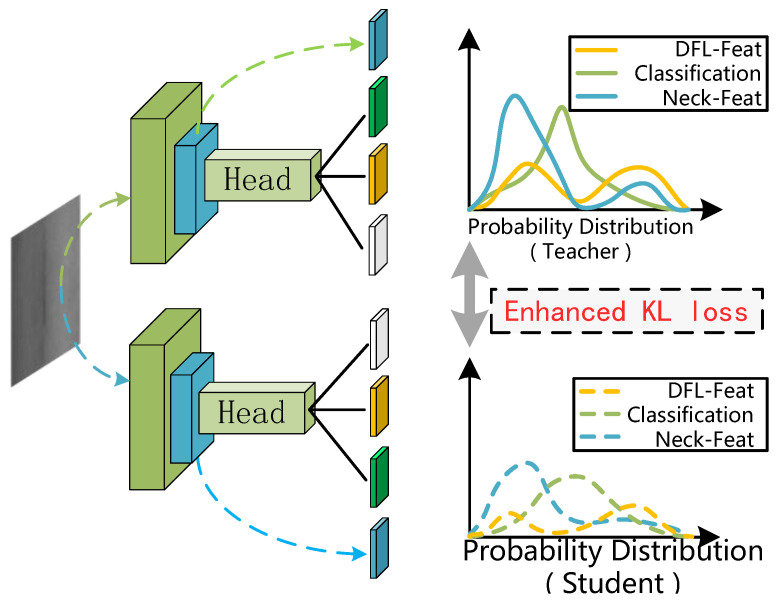
Overview of the self-distillation strategy. The student model learns from the teacher by matching features from the neck, classification, and DFL regression branches. Three branches use an enhanced KL divergence loss. The IoU regression branch is not distilled.

For the features from the AES-FPN fusion network, it can be used to directly compute the distillation loss. For head features, it can be utilized to decouple classification and regression branches. The classification branch distills the classification outputs from both the teacher and student models directly. In the regression branch, DFL provides a more accurate expression of the bounding box distribution by the method of distance distribution, therefore, the distillation operation can only be applied to the DFL component. The additional components, which regress the bounding box through the manipulation of Intersection over Union (IoU), are excluded from the distillation process. The distillation strategy is shown in Figure 7.

Compared with the general distillation model, our distillation model does not need to find an additional teacher model, and the student model is able to achieve better performance beyond the teacher model. Furthermore, we incorporate the feature output from the neck component into the distillation process and effectively prevent information loss during the learning phase. In this framework, the student model is jointly trained using the pseudo labels generated by the pre-trained teacher model and the true labels from the dataset.

### 3.4. Loss Function

The total loss of the SD-GASNet detector comprises the loss of the student model relative to the true labels of the dataset and the loss relative to the pseudo labels of the teacher model. In this paper, the teacher model is the trained student model itself. By minimizing the distillation loss, the student model achieves a significant improvement in detection performance without compromising inference speed. In this subsection, the enhanced KL divergence loss function is first introduced, and then the total loss from the whole training process is given a detailed description.

#### 3.4.1. Enhanced KL Divergence Loss

In machine learning, the KL divergence is often used to quantify the discrepancy between two probability distributions. For example, it is used as part of the loss function in generative models or as a similarity measure in clustering and classification problems. In the field of knowledge distillation, the KL divergence loss is also used to measure the difference between the output distributions of the teacher model and the student model, as illustrated in the following equation.

(23) $$D_{KL}\left(P||Q\right)=\sum_{i=1}^{N}P_{x_{i}}\cdot log\frac{P_{x_{i}}}{Q_{x_{i}}}$$

where $D_{KL}\left(P||Q\right)$ is the KL divergence, P $\in R^{N}$ and Q $\in R^{N}$ denote the original and predicted probability distributions of dimension N, respectively. From the above formula, we know that the KL divergence consists of the self-entropy of *P* and the cross-entropy between *P* and *Q*.

(24) $${D_{KL}}^{e}\left(P||Q\right)=\alpha \sum_{i=1}^{N}P_{x_{i}}\cdot log\frac{P_{x_{i}}}{Q_{x_{i}}}$$

(25) $${D_{KL}}^{e}\left(P||Q\right)=\alpha \sum_{i=1}^{N}P_{x_{i}}\cdot logP_{x_{i}}-\alpha \sum_{i=1}^{N}P_{x_{i}}\cdot logQ_{x_{i}}$$

where ${D_{KL}}^{e}\left(P||Q\right)$ is the enhanced KL divergence loss, α is the weight coefficient that decays according to a cosine annealing schedule throughout the training process. α simultaneously constrains the information entropy of the distribution of the student model and the cross-entropy between the two distributions, facilitating the effective transfer of knowledge from the teacher model to the student model.

Moreover, the decay of the distillation weight benefits the performance improvement of the student model. Throughout the training process, α gradually decays from 1 to 0 using cosine annealing. In the early stages of training, when α is close to 1, resulting in a significant contribution from distillation loss, which helps the parameters of the student model stabilize quickly. In the later stages of training, when the parameters of the model have stabilized, α approaches 0 to allow the own loss of the student model to dominate and thereby promote performance breakthroughs.

#### 3.4.2. The Total Loss of SD-GASNet

The overall loss of SD-GASNet comprises three main supervised loss components: a classification loss ($L_{cls}$), a regression loss ($L_{reg}$), and a feature distillation loss ($L_{feat}$). The regression loss itself is a combination of Distribution Focal Loss ($L_{dfl}$) and GIoU loss ($L_{iou}$). As represented in the following equation:

(26) $$L_{total}=L_{cls}+L_{iou}+L_{dfl}+L_{feat}$$

First, except for the IoU branch, the remaining three branches are distilled between the output of the teacher model and the predictions of the student model using the enhanced KL loss in this paper. The formula is as follows:

(27) $$L_{distill}={D_{KL}}^{e}(P_{s}^{c}\left|\right|Q_{t}^{c}),c\in \left(cls,dfl,feat\right)$$

where $D_{KL}^{e}(P_{s}^{c}\left|\right|Q_{t}^{c})$ is the improved KL divergence loss, *c* corresponds to three different branches, *P* and *Q* are the predicted output of the student model and the teacher model, respectively. When c=feat corresponds to the distillation computation process of three layers of feature in the AES-FPN output.

(28) $$L_{feat}={D_{KL}}^{e}(P_{s}^{feat}\left|\right|Q_{t}^{feat})$$

Next, we describe the computation of the loss in each of the three remaining branches of total loss. Considering that in defect detection task, the number of defect objects is significantly smaller than that of background objects, leading to a substantial imbalance between positive and negative samples. Traditional classification losses can be challenging to optimize for complex defects. Therefore, we introduce the Varifocal Loss (VFL) to address this issue of imbalance between positive and negative samples, as expressed in the following equation:

(29) $$L_{cls}=\frac{1}{N_{pos}}\sum_{x,y}L_{vfl}\left(O_{x,y}^{s},O_{x,y}^{*}\right)+{D_{KL}}^{e}(P_{s}^{cls}\left|\right|Q_{t}^{cls})$$

where $L_{cls}$ denotes the VFL, $N_{pos}$ represents the number of positive samples, $O_{x,y}^{s}$ designates the classification score of the pixel point (x,y) in the student model, and $O_{x,y}^{*}$ is used to denote the target categorization score of the pixel point (x,y). The ${D_{KL}}^{e}(P_{s}^{cls}\left|\right|Q_{t}^{cls})$ represents the computation of the self-distillation loss that exists in the classification branch.

Due to the high similarity between defect images and backgrounds, defect detection is more challenging than traditional object detection. Therefore, for the regression branch, in addition to relying on GIOU loss to regress the bounding boxes, the DFL branch is also brought in to assist in regressing the bounding boxes. DFL branch is used to compute the distance distribution to represent the target box. Due to this distance distribution, the enhanced KL divergence for knowledge distillation is appropriately introduced. The formula of the regression branch is represented as follows:

Due to the high similarity between defect images and backgrounds, defect detection is more challenging than traditional object detection. Therefore, for the regression branch, we adopt DFL as the primary loss to directly learn the distance distribution of bounding box locations. This allows for a more flexible and accurate representation of object boundaries. To further refine the geometric properties of the predicted boxes, we also incorporate an auxiliary GIoU loss as a regularization term. The enhanced KL divergence is then applied to the DFL component for knowledge distillation. The complete formula for the regression branch is represented as follows:

(30) $$L_{reg}=\frac{1}{N_{pos}}\sum_{z}1_{s_{z}^{*}>0}\left(L_{giou}+L_{dfl}\right)+{D_{KL}}^{e}(P_{s}^{dfl}\left|\right|Q_{t}^{dfl})$$

where $N_{pos}$ denotes the number of positive samples, and $1_{s_{z}^{*}>0}$ is the indicator function that is 1 when $s_{z}^{*}>0$, and 0 otherwise, which means that only positive samples contribute to the regression loss calculation. $L_{dfl}$ represents the primary Distribution Focal Loss used to learn the boundary distributions, and $L_{giou}$ represents the auxiliary GIoU loss that provides geometric regularization. The ${D_{KL}}^{e}(P_{s}^{dfl}\left|\right|Q_{t}^{dfl})$ denotes the knowledge distillation of the distance distributions between the student and teacher models, which can further assist in the bounding box regression.

## 4. Experimental Results and Analysis

In this section, first, three public defect datasets are introduced, along with experimental details and evaluation metrics. Subsequently, comparative experiments are carried out to validate the performance of the proposed SD-GASNet method. Finally, ablation experiments are conducted to verify the effectiveness of the proposed model in various aspects.

### 4.1. Experimental Setups

**Datasets Description:** In our experiments we utilize three industrial defect detection datasets: NEU-DET, TILDA, and PCB, all publicly available for surface defect detection tasks. The NEU-DET [44] dataset comprises steel surface defects with six classes: Cr (crazing), Ic (inclusion), Ps (pitted surface), Rs (rolled-in scale), Sc (scratches), and Pc (patches). The dataset includes train and validation sets, totaling 1800 images. The training set consists of 1440 images, while the validation set contains 360 images. The TILDA [45] dataset focuses on textile defect detection with five classes: textile-defects, hole, objects, oil spot, and thread error. This dataset includes 400 images, with 320 images for training and 80 images for validation. The PCB [46] dataset is designed for printed circuit board defect detection with six classes: m_h (missing hole), m_b (mouse bite), o_c (open circuit), sh (short), sp (spur), and s_c (spurious copper). The dataset comprises 693 images, where 593 images are reserved for training and 100 images for validation.
**Implementation Details:** We implement SD-GASNet in PyTorch (version 1.13.0) and conduct all experiments on an NVIDIA A30 GPU (NVIDIA, Santa Clara, CA, USA). The SD-GASNet architecture comprises an LVT (Lightweight Vision Transformer) backbone for feature extraction, an AES-FPN neck for multi-scale feature fusion, and a self-distilled decoupled head (SD-Head) for object detection. Input images are resized to 640 × 640 and predictions are generated at three scales via an anchor-free detection mechanism. During training, comprehensive data augmentation—including HSV color-space jittering, random affine transformations, and mosaic augmentation—is applied to improve model robustness and generalization.
**Parameters Setting:** We train our model for 180 epochs using an AdamW optimizer with a weight decay of 0.05. The initial learning rate is set to 2 × 10^−3^, which begins with a 30-epoch linear warm-up and is then reduced via a cosine annealing schedule to 1% of its initial value. Training is conducted with a batch size of 32. To enhance model stability and generalization, we employ an Exponential Moving Average (EMA) strategy with a decay rate of 0.999. We do not use gradient accumulation or any Test-Time Augmentation (TTA) during evaluation. All models are initialized with COCO pre-trained weights. Training proceeds in two stages: standard training to obtain the teacher model, then distillation training to yield the final model.
**Evaluation Metrics:** We assess the performance of our model using mainstream object detection metrics based on the COCO evaluation protocol. To avoid ambiguity, we adopt a unified naming convention: the primary metric, Mean Average Precision over IoU thresholds from 0.5 to 0.95, is referred to as mAP@[.5:.95] (denoted as AP in our tables). The metric at a single IoU threshold of 0.5 is referred to as mAP@.5 (denoted as AP_50_ in our tables). We also report AP at an IoU of 0.75 (AP_75_) and for objects of different scales (${\mathrm{AP}}_{s}$, ${\mathrm{AP}}_{m}$, ${\mathrm{AP}}_{l}$). Real-time performance is measured by Frames Per Second (FPS), and model complexity is quantified by parameters (Params) and computational cost (GFlops).

### 4.2. Comparisons with State-of-the-Art Methods

To validate the performance of the proposed method, we evaluated SD-GASNet on the NEU-DET, PCB, and TILDA datasets and compared its performance with other representative or State-Of-The-Art (SOTA) detectors, including Faster-RCNN [22], Cascade-RCNN [47], ATSS [48], GFL [43], RetinaNet [49], FCOS [50], ETDNet [4], SSA-YOLO [51], GC-Net [52], DFP-YOLO [53], Wu‘s work [54] and BiContext [9]. Among the selected models, most of them utilize the traditional CNN-based ResNet50 as their backbone, whereas ETDNet and our proposed SD-GASNet employ Transformer-based backbones. For the models we reproduced (marked with * in the tables), the official hyperparameters are used for implementation. To ensure a fair comparison, all data are processed using the same preprocessing methods and split consistently. Detection speed (FPS) is also tested on the same device. In Table 1, Table 2 and Table 3, (*) indicates results reproduced by us based on source code, with all hyperparameters set according to the original paper. First- and second-place results are indicated in red and blue, respectively.

The comparison results are presented in Table 1, Table 2 and Table 3, respectively. Results on NEU-DET Dataset are shown in Table 1: SD-GASNet achieves superior performance across all key metrics, while maintaining highly competitive computational efficiency. Specifically, the core accuracy metrics AP, AP_50_, and AP_75_ reach 49.6, 81.8, and 51.4, respectively. Among all compared models, ETDNet achieves the second highest AP of 45.2, while FCOS achieves the second highest AP_50_ of 79.5. SD-GASNet surpasses them by 4.4% and 2.3% in AP and AP_50_, respectively.

In terms of inference speed, our model reaches 180 FPS. This speed was benchmarked on a single GPU with a batch size of 1 and using FP32 precision. The total end-to-end latency of 5.53 ms per image is composed of model inference (5.07 ms), DFL decoding (0.04 ms), and NMS post-processing (0.42 ms), corresponding to a calculated speed of 180 FPS. Within the scope of the models compared in Table 1, this significantly outperforms the second fastest, SSA-YOLO at 154 FPS, with an improvement of approximately 16.9%.

Furthermore, SD-GASNet also has the fewest parameters with 6.97 M. Notably, SD-GASNet demonstrates outstanding performance in detecting defects of different sizes, ranking first in AP_*s*_, AP_*m*_, and AP_*l*_, showcasing its strong capability in handling multi-scale defects.

Results on PCB Dataset are shown in Table 2: On the PCB dataset, SD-GASNet exhibits excellent performance. Its AP reaches 60.5, significantly leading second-place SSA-YOLO 59.6 by 0.9%. Currently, SD-GASNet achieves the best results in AP with 99.6, AP_75_ with 70.3, AP_*m*_ with 61.2, and AP_*l*_ with 67.8. ATSS performs best in the AP_*s*_ with 35.60, SD-GASNet shows clear advantages in overall performance and speed. It reaches an inference speed of 160 FPS, significantly surpassing SSA-YOLO’s 121 FPS, representing an improvement of approximately 32.2%. The model maintains an extremely low computational footprint, with only 6.97 M parameters and 19.41 GFLOPs.

**Figure 8:**
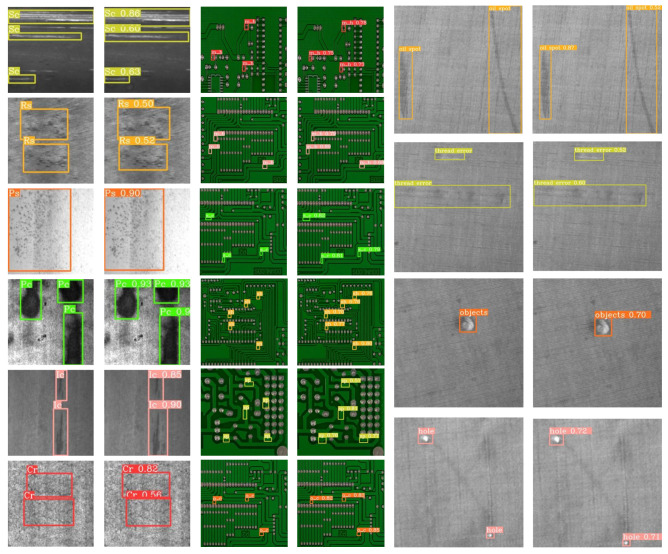
Detection examples by SD-GASNet on NEU-DET, PCB, and TILDA datasets, presented in corresponding groups. NEU-DET abbreviations: ‘Cr’ (crazing), ‘Ic’ (inclusion), ‘Ps’ (pitted_surface), ‘Rs’ (rolled-in_scale), ‘Sc’ (scratches), ‘Pc’ (patches). PCB abbreviations: ‘m_h’ (missing_hole), ‘m_b’ (mouse_bite), ‘o_c’ (open_circuit), ‘sh’ (short), ‘sp’ (spur), ‘s_c’ (spurious_copper).

Results on TILDA Dataset are shown in Table 3: On the more challenging TILDA dataset, SD-GASNet continues to outperform other methods, achieving an AP of 63.1–4.3 percentage points higher than the second-place SSA-YOLO (AP = 58.8). SD-GASNet also achieves the top performance in several key metrics, including AP_50_ (91.7), AP_75_ (72.1), AP_*m*_ (61.1), and AP_*l*_ (67.5). Although SSA-YOLO performs better in AP_*s*_ (65.4 vs. 45.8), a deeper analysis suggests this specific discrepancy stems from a confluence of our model’s intrinsic design and the dataset’s unique properties. Fundamentally, our FGPEM module is optimized for structured frequency-domain features, but tiny defects degrade into non-structural, pulse-like signals after aggressive downsampling, making them difficult for our filter to capture. This is then critically exacerbated by TILDA’s dense textile background, which creates a dominant, high-frequency noise floor that masks these weak signals. Despite this specific challenge with small objects, SD-GASNet demonstrates a superior overall balance between accuracy and efficiency. It achieves a higher mAP of 63.1, with fewer parameters (6.97 M) and a significantly faster inference speed (162 FPS vs. 154 FPS). Detailed visualizations of the detection results on the aforementioned datasets are presented in Figure 8.

### 4.3. Ablation Studies

To analyze the effectiveness of each proposed module in SD-GASNet, comprehensive ablation experiments are implemented on the NEU-DET dataset. The detection performance of different modules is evaluated using AP and AP_50_ metrics. Additionally, the efficiency of the model is measured in terms of Params, GFLOPs, and FPS.

**Analysis of FIGA mechanism and CS module:** To evaluate the effectiveness of the FIGA mechanism and the CS module, the ablation results are shown in Table 4. The experiment started with a baseline model that contained no additional modules. It achieved an AP of 44.4 and an ${AP}_{50}$ of 78.3, with 5.45 M parameters and a computational cost of 16.65 GFLOPs. Introducing the FIGA mechanism resulted in significant performance improvements, increasing AP by 2.3 points to 80.6 and ${AP}_{50}$ by 3.9 points to 48.3. This demonstrates that the FIGA mechanism effectively enhances spatial-domain feature representation by processing multi-scale aggregated features from a frequency-domain perspective. In contrast, introducing the CS module alone yielded limited effectiveness. The improvements in AP and ${AP}_{50}$ were only 1.1 and 0.9 points, respectively, while also increasing the computational load.

This outcome aligns with expectations, as the CS module is designed primarily to work in conjunction with the FIGA mechanism. It aims to address the increased complexity of the feature and the potential loss of top-level information introduced by FIGA. Finally, combining the FIGA and CS modules achieves the best overall performance with an AP of 81.8 and an AP_50_ of 49.6. This result further validates the effectiveness of the FIGA mechanism and the synergistic advantage of integrating it with the CS module, despite the combination incurring the highest computational overhead (6.97 M parameters and 19.41 GFLOPs).

**Table 4:** Analysis of FIGA mechanism and CS module. Parameter counts (Params) are in millions (M). All Average Precision (AP) metrics are reported in percentage (%). Bold values indicate the best performance in each column.

FIGA	CS	Params ↓	GFLOPs ↓	FPS ↑	AP	AP_50_
No	No	**5.45 M**	**16.65**	**187**	44.4	78.3
No	Yes	6.24 M	17.78	192	45.3	79.4
Yes	No	5.99 M	18.32	182	48.3	80.6
Yes	Yes	6.97 M	19.41	180	**49.6**	**81.8**

**Analysis of PDCT Frequency Selection Strategy:** To empirically validate the key design choices within our PDCT filter, we conducted two further ablation studies. A crucial finding is that these design choices exclusively affect detection accuracy. The model’s computational costs (6.97 M Params, 19.41 GFLOPs, 180 FPS) remain constant regardless of the grid size or the specific frequencies selected. This is because these choices only influence the pre-computed initial values within the PDCT filter, not its size or the subsequent operations.

First, we analyzed the impact of the frequency quantization grid size, as shown in Table 5. We started our analysis by considering the 7 × 7 grid, a classic choice in frequency-domain methods like JPEG compression and FcaNet [13], which achieved a solid AP of 48.7. To better adapt to the subtle and complex frequency patterns of industrial defects, we explored expanding this grid. Increasing the grid size to 10 × 10 resulted in a significant performance improvement, boosting the AP to a peak of 49.6 and the AP_50_ to 81.8. This demonstrates that a finer frequency resolution is indeed beneficial for capturing critical defect-related details. However, this trend did not continue indefinitely. Further increasing the grid size to 15 × 15 and 20 × 20 led to a gradual decline in performance across all metrics. This suggests that overly fine grids may introduce sensitivity to irrelevant high-frequency noise, ultimately hindering generalization. Therefore, this result validates our choice of a 10 × 10 grid as the optimal trade-off between frequency resolution and robust feature representation for our task.

Second, we evaluated the sensitivity to the number of selected frequency components, as detailed in Table 6. Increasing the number of components from 4 (low2 + hig2) to 8 (low4 + hig4) resulted in a significant performance gain, with the AP improving from 48.5 to 49.6, AP_50_ from 80.8 to 81.8, and AP_75_ from 49.5 to 51.4. This demonstrates the benefit of incorporating a richer set of frequency information. However, this trend reverses when more components are added. Increasing the count to 16 (low8 + hig8) caused the AP to drop to 49.2, and a further increase to 32 components led to a more significant decline to an AP of 48.2. This performance degradation suggests that an excessive number of frequency components may introduce less discriminative or even irrelevant frequency patterns, which can interfere with the final feature representation. Thus, the 8-component configuration is empirically validated as the optimal choice, achieving the best performance across all key metrics.

**Analysis of Multi-level Feature Fusion Module:** To demonstrate the effectiveness of AES-FPN that combines the FIGA mechanism and the CS module, we present the results of the comparison with FPN, PAFPN, and CM-FPN in Table 7. The results show that AES-FPN achieves the best performance among all compared methods, with the highest AP of 81.8 and AP_50_ of 49.6. In comparison, CM-FPN achieves the second highest AP_50_ of 45.1, which can be attributed to its channel modulation mechanism that enriches features representation at the output channel level. PAFPN ranks second in AP with 79.1, benefiting from its ability to capture information from both cross-layer and specificity feature through top-down and bottom-up feature fusion operations. In terms of parameter scale, PAFPN has the smallest model size, with only 5.58 M parameters and 15.58 GFLOPs. By contrast, AES-FPN ranks second in this aspect, with 6.97 M parameters and 19.41 GFLOPs.

**Figure 9:**
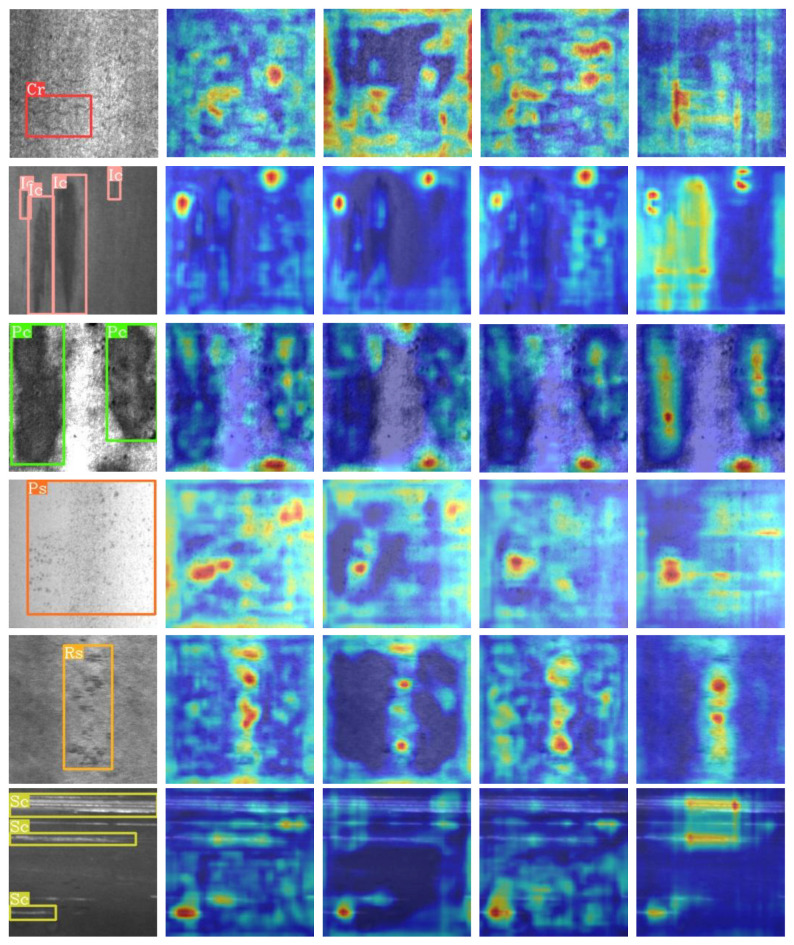
Visualization of attention maps produced by various feature fusion networks on the NEU-DET dataset, where red regions indicate higher attention values. The images, arranged from left to right, include the original image, followed by results obtained using FPN, PAFPN, CMFPN, and the proposed AES-FPN. For each defect class, a single sample image is randomly selected for comparative analysis.

AES-FPN achieves the best results, which can be attributed to the two proposed mechanisms. The FIGA mechanism improves the interaction between the cross-layer feature through MFA and FGPEM, and utilizes information from the frequency domain to enhance the feature representation of spatial domain. This enables the model to better perceive boundary and texture information. The CS module addresses the problem of channel information loss in high-level features, providing ample feature information for detection and distillation. Figure 9 shows a qualitative comparison of the heatmap results of feature fusion among the different methods discussed above.

**Table 7:** Analysis of Multi-level Feature Fusion Module. Parameter counts (Params) are in millions (M). All Average Precision (AP) metrics are reported in percentage (%). Bold values indicate the best performance in each column.

Type of Neck	Params ↓	GFLOPs ↓	FPS ↑	AP	AP_50_
FPN	10.87 M	25.3	173	43.6	77
PAFPN	**5.58 M**	**15.58**	176	43.9	79.1
CM_FPN	10.86 M	25.62	172	45.1	77.8
AES-FPN (ours)	6.97 M	19.41	**180**	**49.6**	**81.8**

**Analysis of Distillation Loss:** To validate the effectiveness of the enhanced KL divergence loss, three other loss functions of knowledge distillation are selected for comparison, namely Mean Squared Error (MSE), Cross-Entropy (CE), and the traditional KL divergence loss (KL). The NEU-DET dataset is used to evaluate the performance of these four loss functions. As shown in Table 8, our improved KL divergence loss obtains the best overall performance in several key metrics, yielding the highest AP of 81.8, AP_50_ of 49.6, AP_75_ of 51.4, and AP_*l*_ of 59.5. Compared with the standard KL divergence loss, the enhanced version improves AP by 1.5 points and AP_50_ by 1.1 points. Meanwhile, MSE demonstrates superior performance in terms of AP_*s*_ and AP_*m*_, achieving 39.5 and 44.7, respectively.

**Table 8:** Comparison of different loss functions. All Average Precision (AP) metrics are reported in percentage (%). Bold values indicate the best performance in each column.

Type of Loss	AP	AP_50_	AP_75_	AP_*s*_	AP_*m*_	AP_*l*_
KL	48.5	80.3	50.2	37.4	43.3	59.2
CE	46.4	79.7	49.7	36.9	43.4	58.1
MSE	48.3	80.9	50.8	**39.5**	**44.7**	58.7
${D_{KL}}^{e}$ (ours)	**49.6**	**81.8**	**51.4**	38.2	44.1	**59.5**

**Effective Analysis of Neck Feature Distillation:** Traditional knowledge distillation in object detection typically focuses solely on the output of the detection head (‘Head KD’). In this paper, we investigate the benefits of additionally distilling intermediate features from the feature fusion neck (‘Neck KD’). The results presented in Table 9 demonstrate the effectiveness of this extended approach. Compared with distilling only the head output, incorporating features from the neck module further improves performance across several metrics. Specifically, AP increases by 0.4 points, AP_50_ by 1.0 points, and AP_75_ by 0.4 points. These results demonstrate that the integration of neck features into the knowledge distillation process is both reasonable and effective to enhance the performance of the student model.

The FIGA mechanisms employed in our feature fusion process generate sophisticated feature representations. Distilling only the final output of the detection head may not provide sufficient guidance for the student network to capture the intricate fusion patterns learned by the teacher. By incorporating neck features into the distillation process, we provide more detailed layer-wise supervision, enabling the student to better replicate the teacher model fusion strategy and achieve improved performance, particularly in terms of AP, AP_50_, AP_75_, AP_*s*_ and AP_*l*_.

**Table 9:** Ablation study comparing knowledge distillation strategies. ‘Baseline’ represents the model trained without knowledge distillation. ‘Head KD’ distills only the head output features, while ‘Neck KD’ distills both intermediate neck features and head output features. All Average Precision (AP) metrics are reported in percentage (%). Bold values indicate the best performance in each column.

Method	AP	AP_50_	AP_75_	AP_*s*_	AP_*m*_	AP_*l*_
Baseline	48.2	79.7	50.2	36.9	43.5	58.2
Head KD	49.2	80.8	51.0	37.7	**44.5**	58.8
Neck KD (ours)	**49.6**	**81.8**	**51.4**	**38.2**	44.1	**59.5**

**Comparison With Real-Time Detectors:** To explore the performance of our model in terms of speed and accuracy, comparative experiments are conducted on the NEU-DET dataset with several state-of-the-art real-time detectors, including RT-DETR [55], YOLOX [56], YOLOV8, YOLOV9 [57], and YOLOV10 [58]. Table 10 shows the results of the different detectors. SD-GASNet surpasses RT-DETR-l by 6.1% in AP and 7.9% in AP_50_ and outperforms YOLOv10s by 4.4% in AP and 6.2% in AP_50_. It also achieves the lowest model complexity in terms of Params and GFLOPs. However, its FPS is lower than that of the YOLO series detectors. This is likely primarily due to the incorporation of the ViT module, which results in a slower inference speed compared to CNN-based models. However, SD-GASNet still achieves an inference speed of 180 FPS, surpassing RT-DETR and demonstrating a favorable trade-off between detection accuracy and inference speed.

**Table 10:** Comparison with newest Real-Time Detectors. Parameter counts (Params) are in millions (M). All Average Precision (AP) metrics are reported in percentage (%). Bold values indicate the best performance in each column.

Model	Params	GFLOPs	FPS	AP	AP_50_	AP_75_
RT-DETR-L	63 M	103	88	43.5	73.9	44.7
YOLOXs	8.94 M	26.65	144	43.9	77	44.1
YOLOv8s	11.1 M	28.4	**269**	44.0	75.5	46.0
YOLOv9s	15.2 M	26.7	211	45.3	77.1	48.9
YOLOv10s	15.77 M	24.5	211	45.2	75.6	47.4
Ours	**6.97 M**	**19.41**	180	**49.6**	**81.8**	**51.4**

## 5. Conclusions

In summary, the proposed SD-GASNet has achieved significant results in the field of industrial surface defect detection. It successfully achieves a balance between efficiency and high performance by using a lightweight feature extractor named MLVT, an improved feature fusion network called AES-FPN, and self-distillation training based on an enhanced KL divergence loss. SD-GASNet exhibits excellent performance and good generalization ability on multiple datasets, such as NEU-DET, PCB, and TILDA. In addition, its inference speed is significantly faster than other SOTA models, making it particularly suitable for industrial inspection environments.

However, the experimental results also reveal certain limitations of SD-GASNet in detecting small objects, particularly on datasets with highly textured backgrounds. As analyzed in Section 4.2, this challenge stems from a combination of our frequency-based enhancement module’s inherent bias towards structured features and the loss of structural integrity of tiny objects on deep feature maps. Therefore, in future work, it is necessary to further optimize the small object detection capabilities of SD-GASNet, with potential directions including the development of a scale-aware frequency enhancement mechanism or the integration of lightweight spatial attention modules at earlier stages of the network.

## Figures and Tables

**Table 1:** Quantitative comparisons of different methods on the NE-DET dataset. Parameter counts (Params) are in millions (M). All Average Precision (AP) metrics are reported in percentage (%).

Model	Backbone	Params	GFLOPs	FPS	AP	AP_50_	AP_75_	AP_*s*_	AP_*m*_	AP_*l*_
(*)Faster-Rcnn	ResNet50	41.37 M	90.92	45	41.4	77.7	39.5	32.0	36.3	47.4
(*)Cascade-Rcnn	ResNet50	69.23 M	118.77	28	43.3	75.7	45.4	32.3	37.3	49.0
(*)ATSS	ResNet50	32.13 M	80.57	49	43.0	76.1	43.9	31.8	36.4	52.4
(*)GFL	ResNet50	32.27 M	81.81	49	42.8	74.5	41.9	30.6	36.2	50.3
(*)RetinaNet	ResNet50	36.43 M	82.45	56	41.3	77.2	38.9	30.4	34.1	48.3
(*)FCOS	ResNet50	32.13 M	123.05	49	42.5	79.5	40.6	31.2	38.4	50.7
(*)ETDNet	MLVT	7.01 M	23.91	72	45.2	77.7	44.5	34.8	38.8	56.3
(*)SSA-YOLO	-	12.51 M	17.61	154	42.2	74.9	41.7	37.3	36.5	57.8
GC-Net	-	-	-	-	42.4	77.1	42.0	36.3	33.4	52.4
DFP-YOLO	DarkNet	8.92 M	-	125	-	73.8	-	-	-	-
Wu et al. [54]	DarkNet	-	-	-	-	78.2	-	-	-	-
Ours	MLVT	6.97 M	19.41	180	49.6	81.8	51.4	38.2	44.1	59.5

**Table 2:** Comparison results of different methods on PCB dataset. Parameter counts (Params) are in millions (M). All Average Precision (AP) metrics are reported in percentage (%).

Model	Backbone	Params	GFLOPs	FPS	AP	AP_50_	AP_75_	AP_*s*_	AP_*m*_	AP_*l*_
(*)Faster-Rcnn	ResNet50	41.38 M	75.65	39	53.70	95.3	54.6	23.20	54.5	55.4
(*)Cascade-Rcnn	ResNet50	69.24 M	103.51	29	53.6	95.4	54.3	24.5	54.3	53.6
(*)ATSS	ResNet50	32.13 M	64.62	47	54.50	96.1	53.9	35.60	55.1	54.6
(*)GFL	ResNet50	32.27 M	65.61	46	54.6	95.2	55.8	22.8	55.2	58.4
(*)RetinaNet	ResNet50	36.45 M	66.26	50	52.4	94.8	52.7	27.3	52.9	55.4
(*)FCOS	ResNet50	32.13 M	63.1	48	51.0	91.9	49.8	20.3	51.3	54.9
(*)ETDNet	MLVT	7.01 M	23.91	61	57.8	95.9	64.5	22.4	58.6	58.6
(*)SSA-YOLO	-	12.51 M	17.6	121	59.6	98.3	63.8	28.9	59.6	58.3
BiContext	DarkNet	28.3 M	104.9	-	56.3	97.9	-	-	-	-
Ours	MLVT	6.97 M	19.41	160	60.5	99.6	70.3	27.6	61.2	67.8

**Table 3:** Comparison results of different approaches on TILDA dataset. Parameter counts (Params) are in millions (M). All Average Precision (AP) metrics are reported in percentage (%).

Model	Backbone	Params	GFLOPs	FPS	AP	AP_50_	AP_75_	AP_*s*_	AP_*m*_	AP_*l*_
(*)Faster-Rcnn	ResNet50	41.37 M	67.82	47	39.20	66.1	43.5	47.00	50.2	33.6
(*)Cascade-Rcnn	ResNet50	69.21 M	95.66	28	32.5	59.3	30.6	32.4	41.1	29.4
(*)ATSS	ResNet50	32.12 M	56.4	51	52.00	80.3	58.1	45.90	51.0	54.3
(*)GFL	ResNet50	32.27 M	57.27	51	54.4	80.8	59.5	59.1	56.6	53.7
(*)RetinaNet	ResNet50	36.41 M	57.61	57	50.3	77.4	51.6	53.2	50.3	51.5
(*)FCOS	ResNet50	32.13 M	55.07	50	54.4	83.0	58.1	59.7	55.5	52.7
(*)ETDNet	MLVT	7.01 M	23.91	27	58.7	86.6	67.2	60.7	52.5	61.0
(*)SSA-YOLO	-	12.51 M	17.6	154	58.8	88.6	68.5	65.4	53.5	65.6
Ours	MLVT	6.97 M	19.41	162	63.1	91.7	72.1	45.8	61.1	67.5

**Table 5:** Ablation study on the PDCT frequency quantization grid size. All Average Precision (AP) metrics are reported in percentage (%). Bold values indicate the best performance in each column.

Grid Size	AP	AP_50_	AP_75_
5 × 5	48.0	80.4	49.2
7 × 7	48.7	80.9	50.8
**10 × 10**	**49.6**	**81.8**	**51.4**
15 × 15	49.1	81.2	51.1
20 × 20	48.9	80.2	50.5

**Table 6:** Ablation study on the number of selected frequency components. All Average Precision (AP) metrics are reported in percentage (%). Bold values indicate the best performance in each column.

Configuration	Frequencies	AP	AP_50_	AP_75_
low2 + hig2	4	48.5	80.8	49.5
low**4** + hig**4**	**8**	**49.6**	**81.8**	**51.4**
low8 + hig8	16	49.2	81.1	50.6
low16 + hig16	32	48.2	79.6	49.3

## Data Availability

The code and data are publicly available in the following repository: https://github.com/wwbing/GASNet (accessed on 1 December 2025).
